# Real-Time Analysis on Drug-Antibody Ratio of Antibody-Drug Conjugates for Synthesis, Process Optimization, and Quality Control

**DOI:** 10.1038/s41598-017-08151-2

**Published:** 2017-08-10

**Authors:** Yubo Tang, Feng Tang, Yang Yang, Lei Zhao, Hu Zhou, Jinhua Dong, Wei Huang

**Affiliations:** 1 Key Laboratory of Structure-Based Drug Design and Discovery, Ministry of Education, Shenyang Pharmaceutical University, Shenyang, 110016 China; 2 CAS Key Laboratory of Receptor Research, CAS Center for Excellence in Molecular Cell Science, Shanghai Institute of Materia Medica, Chinese Academy of Sciences, 555 Zuchongzhi Road, Pudong, Shanghai, China 201203

## Abstract

Drug-antibody ratio (DAR) of antibody-drug conjugates (ADCs) is important for their therapeutic efficacy and pharmacokinetics, therefore control on DAR in synthesis process is a key for ADC quality control. Although various analytical methods were reported, the real-time monitoring on DAR is still a challenge because time-consuming sample preparation is usually needed during the analysis. Antibody deglycosylation of ADC simplifies DAR measurement, however long-time PNGaseF digestion for deglycosylation hampers the real-time detection. Here, we report a rapid DAR analysis within 15 min by robust deglycosylation treatment and LC-MS detection that enables real-time DAR monitoring for optimization on ADC synthetic process. With this approach, we were able to screen suitable conjugation conditions efficiently and afford the ADCs with expected DARs. To the best of our knowledge, this is the first report on real-time DAR analysis of ADCs for conjugation optimization and quality control, compatible with random lysine-linked ADCs, glycosite-specific ADCs, and the complicated dual-payload ADCs.

## Introduction

Antibody-drug conjugates (ADCs) carry a highly potent small-molecule toxin covalently connected on the antibody via a proper linker^1–3^. For therapeutic ADCs in cancer treatment^4^, the antibody targets specific antigen of tumor cell surface with high binding affinity, thereafter the intact ADC was internalized into the tumor cells with the antigen and digested in the lysosome to release the antitumor toxin^3, 4^. This tumor targeting strategy of ADC successfully improves the drug efficacy and safety^5^, and attracts great research interest during the past decade. Many novel technologies on site-specific conjugation^6–15^, optimal linker^2, 16–18^, new payload^19^, dual-payload strategy^8, 20^, etc., have emerged for new-generation ADC development. Up to date, there are 2 ADC drugs launched on the market and over 40 ADC candidates in clinical trials^21^.

Drug antibody ratio (DAR) is an important parameter of ADC. Low DAR could reduce the antitumor efficacy, while high DAR may affect antibody structure, stability, and antigen binding etc. therefore causing loss of activity^22^. DAR values are also important for therapeutic index of ADCs^23^. In most of ADC drug candidates, their DAR values were maintained at about 2–4. Hence, to control DAR during ADC preparation is a key procedure and comes with an urgent need for real-time DAR analysis on *in situ* ADC samples^24^. Currently, several analytical methods have been reported for DAR measurement including UV/Vis spectroscopy^25^, hydrophobic interaction chromatography (HIC)^26^, RP-HPLC^27^, and LC-MS^28–30^. UV/Vis detection is not compatible with *in situ* ADCs because of the influence of the excess small-molecule reagent in the reaction aliquots. HIC, RP-HPLC, and LC-MS analysis could provide precise DAR characterization on intact or digested ADC samples, however HIC was mainly limited in Cys-linked ADCs^27^ and ADC fragment analysis with RP-HPLC or LC-MS required time-consuming digestion procedure and data processing^27, 30^. LC-MS measurement on intact ADCs demonstrated great potential in the literature for DAR analysis of all kinds of ADCs with ESI-(Q)TOF-MS^8, 29, 31^, native MS^32^, and ion mobility MS^32^, CE-MS^33^, etc. The approach using ESI-(Q)TOF-MS for intact ADCs detection^8, 29, 31^ after Fc deglycosylation is most promising for real-time analysis except the only obstacle of long-time deglycosylation with the glycosidase PNGaseF (peptide-N-glycosidase from *Flavobacterium meningosepticum*)^31^. IgG Fc N-glycans consist of heterogeneous glycoforms that make the DAR analysis more complicated since the lysine-linked ADCs bear heterogeneous numbers of small molecules as well. There are about 20–30 m/z deconvoluted peaks for a typical lysine-linked ADC and the deglycosylation could dramatically simplify the DAR measurement by reducing the m/z to about 5–8 deconvoluted peaks. Therefore, efficient deglycosylation of ADC is a crucial step for real-time DAR analysis. Here, we report a rapid process of deglycosylation and LC-MS determination within 15 minutes for real-time DAR analysis. With this approach, we monitored the ADC conjugation reaction, optimized the conjugation conditions, and controlled the DAR as expected. Random lysine-linked ADCs, glycosite-specific ADCs (gsADCs)^8^, and complicated dual-payload ADCs (dpADCs) were prepared with this real-time analytical method.

## Results and Discussion

### Synthesis of lysine-linked ADCs, glycosite-specific ADCs, and dual-payload ADCs

**Figure 1:**
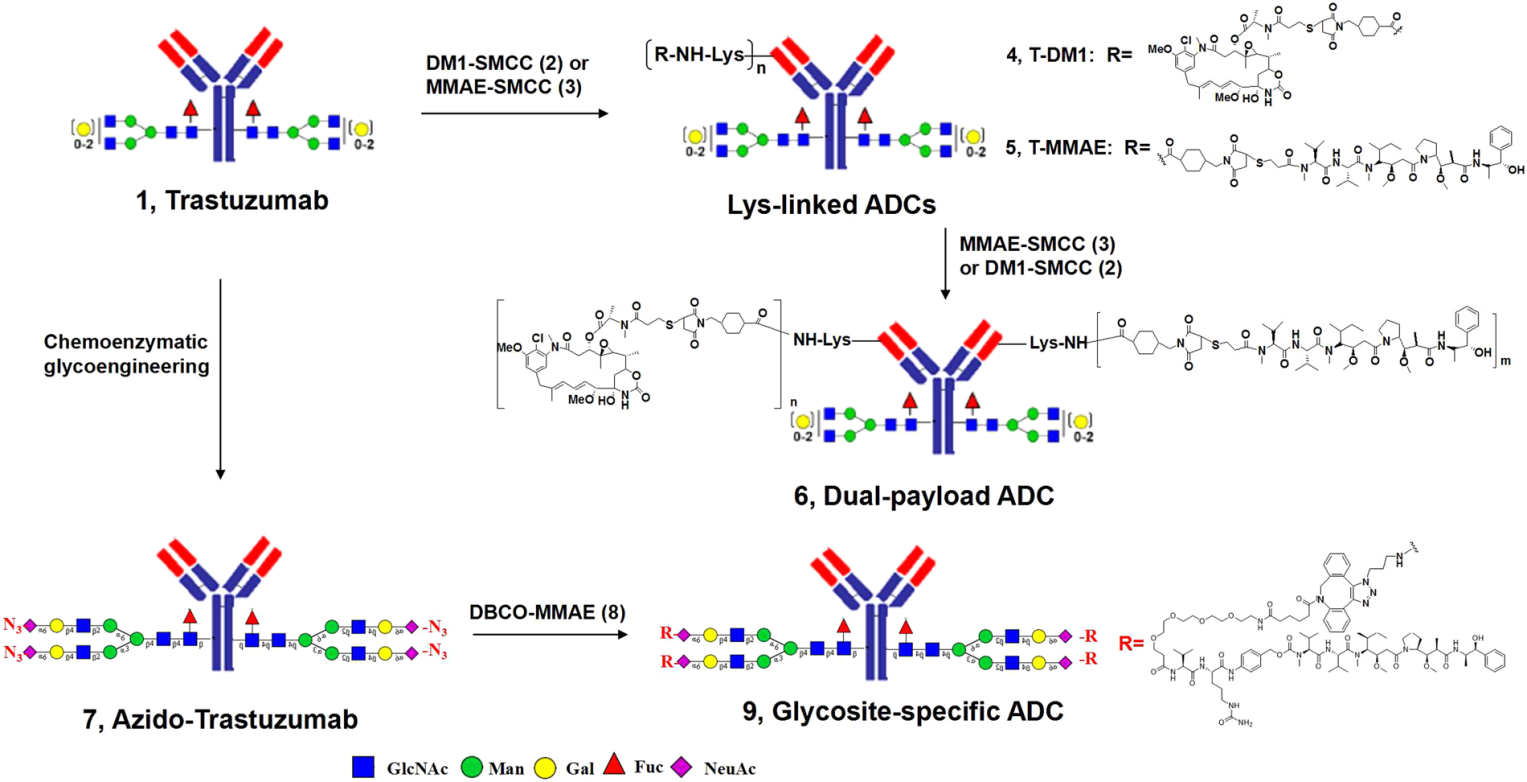
The synthesis of lysine-linked ADCs, dual-payload ADC, and glycosite-specific ADC.

We synthesized the random lysine-linked ADCs (**4** and **5**), dpADC (**6**), and gsADC (**9**) following the reported chemistry with optimization^8, 34^. Two cytotoxins, DM1 (*N*_2_ *′*-deacetyl-*N*_2_ *′*-(3-mercapto-1-oxopropyl)-maytansine) and MMAE (monomethyl auristain E), and the classic linker SMCC (N-succinimidyl 4-(N-maleimidomethyl)cyclohexane-1-carboxylate) were employed. The synthetic route of these ADCs was provided in Fig. 1 and the key intermediates of DM1-SMCC (**2**) and MMAE-SMCC (**3**) were prepared as shown in the supplementary schemes. In short, the drug-linker intermediate of DM1-SMCC was prepared by thiol-ene click reaction of commercial DM1 and SMCC in a neutral buffer (Scheme S1). For MMAE-SMCC, the protected 3-mercaptopropionyl group was introduced onto the N-terminal secondary amine of MMAE by coupling reaction, then, the thiol group was deprotected and conjugated with the maleimide moiety of SMCC to give the target intermediate (Scheme S2). The N-hydroxy-succinimide active ester on DM1-SMCC and MMAE-SMCC randomly reacted with the lysine residues of the antibody herceptin to afford ADC **4** and **5** respectively. The dual-payload ADC (**6**) was obtained by successive conjugation with DM1-SMCC and MMAE-SMCC. The glycosite-specific ADC (**9**) was prepared following our recent published approach of chemoenzymatic glycoengineering on Fc glycosite^8^. As shown in Fig. 1, N-glycosylation of herceptin was remodeled with an azido-containing biantennary N-glycan substrate catalyzed by an Endo-glycosidase mutant in a one-pot strategy^8^. Then, a pre-synthesized DBCO (dibenzoazacyclooctyne)-labeled MMAE was assembled on the azido-glycan via copper-free Huigen cycloaddition reaction^35^ to give gsADC **9**.

### Optimal deglycosylation of ADCs for rapid LC-MS analysis

**Figure 2:**
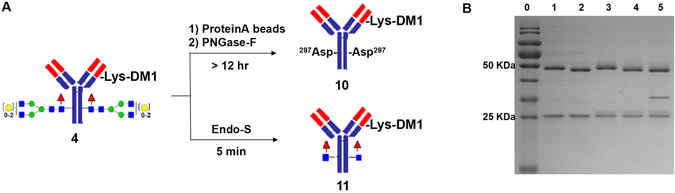
ADC deglycosylation with PNGase-F and Endo-S. A) Schematic procedures for ADC deglycosylation with PNGase-F and Endo-S; B) SDS-PAGE analysis of ADC deglycosylation, lane 0: protein ladder, line 1: commercial herceptin, line 2: deglycosylated herceptin with Endo-S, line 3: ADC **4** (T-DM1), line 4: deglycosylated ADC **4** with Endo-S after 5 mins, line 5: deglycosylated ADC **4** with PNGase-F after overnight.

**Figure 3:**
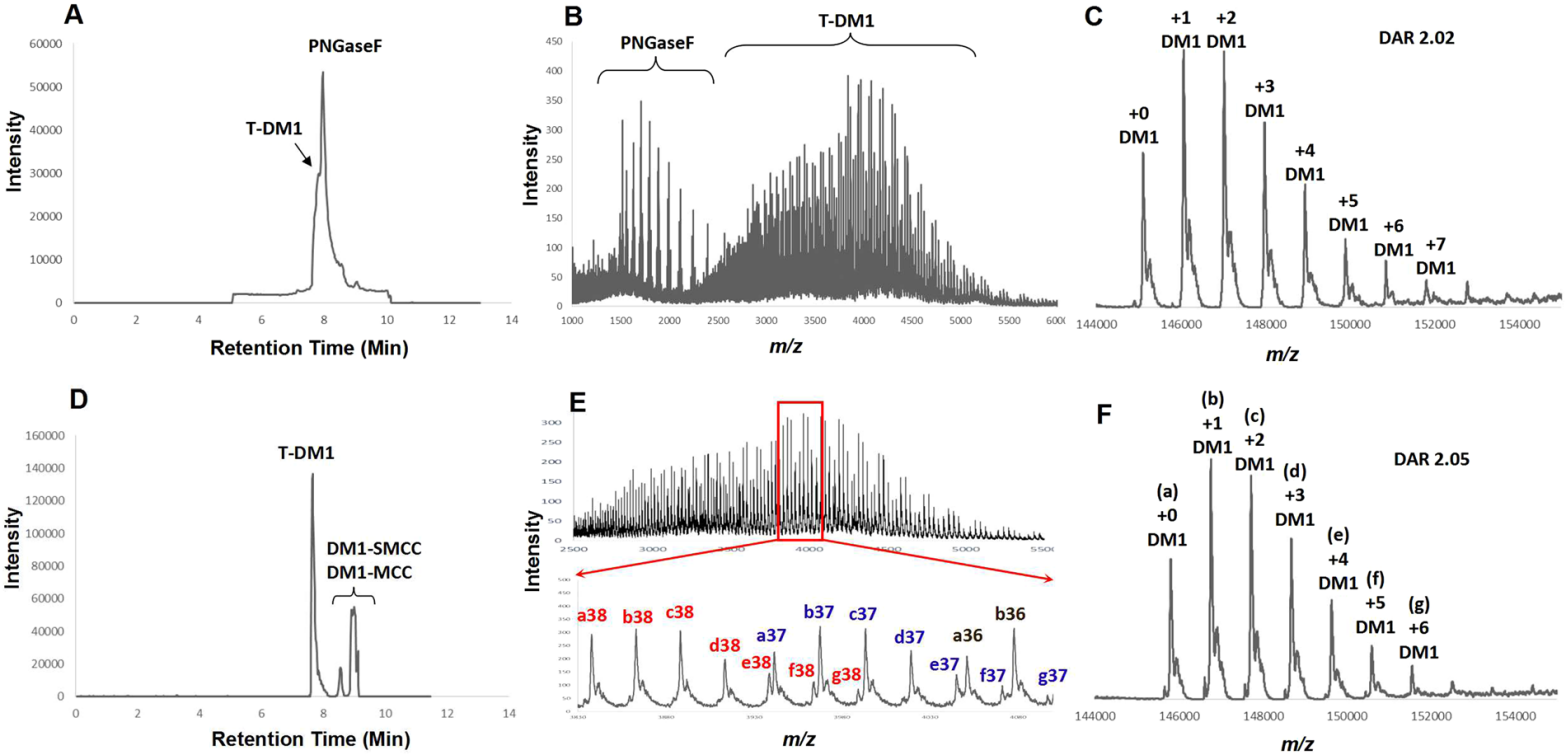
Comparison of LC-MS data of deglycosylated ADC **4** by PNGase-F and Endo-S. Total Ion Chromatograms (TIC) of T-DM1 (**4**) after deglycosylation with PNGase-F (Panel A) and Endo-S (Panel D); multi-charged *m/z* profiles of **4** after deglycosylation with PNGase-F (Panel B) and Endo-S (Panel E, upper: wide mass range 2500–5500; bottom: zoom-in mass range 3800–4100); deconvolution data and DAR calculation of **4** after deglycosylation with PNGase-F (Panel C) and Endo-S (Panel F).

With the prepared ADC samples in hand, we started to develop a rapid LC-MS analysis for DAR detection which could be employed in real-time monitoring. Firstly, we sought to optimize the deglycosylation procedure for ADC analysis. The antibodies in ADC usually carry a conserved N-glycan on Asn297 of the heavy chains and the glyco-form was a heterogeneous mixture mainly containing G0F, G1F, and G2F structures. Therefore, the MS profile of intact ADC consists of dozens of *m/z* values by combination of heterogeneous glycosylation and small-molecule payload numbers that complicated the DAR measurement. In order to simply the determination, deglycosylation of ADC was performed in previous literatures^23, 29^ using a peptide-N-glycosidase from *Flavobacterium meningosepticum* (PNGase F). PNGase F cleaves the amide bond between the first saccharide N-acetylglucosamine (GlcNAc) and the Asn297 side chain to release the free N-glycan from the antibody (Fig. 2A). After deglycosylation, the MS of antibody becomes homogeneous by removal of mixed glycoforms (Figure S1). Accordingly, the MS profiles of ADC (Fig. 3C and F) were simplified with only mixed *m/z* values of different payload numbers. The DAR was then easily calculated as the average payload number based on the sum of all deconvoluted mass intensities.

The deglycosylation of IgG by PNGase-F usually requires long-time treatment (for overnight in the literature) that is not compatible to real-time monitoring. Recently, we have developed an efficient approach^8, 36^ for IgG glyco-remodeling and the rapid deglycosylation activity of an Endo-N-acetylglucosaminidase from *Streptococcus pyogenes* (Endo-S)^37^ inspired us to employ this enzyme for ADC deglycosylation and real-time DAR detection. Fig. 2 showed the comparison of PNGase-F and Endo-S for ADC deglycosylation. Endo-S cleaves the glycan between the GlcNAcβ1,4GlcNAc motif completely within 5 min at a very low enzyme concentration (1.5 μg/mL). SDS-PAGE in Fig. 2B showed the efficient deglycosylation of both IgG (lane 2) and ADC (lane 4) by Endo-S as their heavy chains shifted downside ~2 KDa compared with the bands before deglycosylation (lane 1 and 3). On the other hand, PNGase-F digestion required a higher enzyme concentration (200 μg/mL) and more than 12 hr incubation to complete the deglycosylation. The band around 35 KDa in Fig. 2B lane 5 is the PNGase-F, on the contrary, there is no detectable band of Endo-S around 100 KDa (molecular weight of Endo-S). More importantly, because of long-time digestion by PNGase-F, the drug conjugation of ADC may still be running on and DAR value could increase during the deglycosylation, therefore pre-purification of ADC by protein-A affinity chromatography is needed before the deglycosylation and DAR detection by PNGase-F approach.

The rapid and robust deglycosylation of ADC by Endo-S encouraged us to further compare the DAR determination via LC-MS with the data of PNGase-F treated ADC. In Fig. 3, panel A showed the total ion chromatogram (TIC) profile of PNGase-F digested ADC **4** and both two proteins were observed in TIC and MS detection (panel B) because of the high concentration of the enzyme. The deconvolution data (panel C) indicated a series of MS peaks assigned as ADCs bearing increasing payload numbers and DAR was calculated as 2.02. In comparison, Endo-S treated ADC **4** from the same reaction was directly subject to the LC-MS determination right after 5 min deglycosylation. The TIC profile (panel D) showed the ADC **4** as the only protein portion since the trace amount of Endo-S used for deglycosylation was not detectable. The small molecule reagent (DM1-SMCC) and its derivative without pre-purification in the reaction aliquot were also observed in the TIC figure and were completely separated from the protein portion. Panel E showed the multi-charged *m/z* of the ADC and the individual peaks in the zoom-in mass range (bottom chart) were assigned and marked with their deconvoluted masses (panel F) and charge numbers. The determined DAR of ADC **4** by Endo-S approach was 2.05, in high agreement with the DAR detected by PNGase-F approach, that demonstrated the consistence and reliability of both methods. In these comparison data, Endo-S exhibits obvious advantages in rapid deglycosylation, excellent separation of ADC in chromatography, and efficient detection of reaction aliquots for real-time monitoring.

It was reported that IgG deglycosylation with PNGaseF digestion could complete within 1 hour when incubating at 45 °C^29^. Moreover, a commercial optimal PNGaseF from New England Biolabs, named Rapid^TM^ PNGase-F was able to cleave IgG N-glycans after 10 min incubation at 50 °C (requiring a 2-min pre-treatment at 80 °C in some cases) as described by the manufacturer. The shorten time of deglycosylation makes this PNGaseF a promising enzyme for real-time DAR detection, however the increased temperature (45–50 °C) brings another concern that the DAR values might significant changed during the incubation at a higher temperature. As shown in Figure S2, we did a comparison DAR measurement by incubating the same reaction aliquot samples with Endo-S for 10 mins at 25 °C or 50 °C respectively. Surprisingly, DAR measured after 50°C incubation increased to 5.16 while the DAR measured after 25°C incubation was only 1.54 (Figure S2, panel A and B). Furthermore, decomposition of ADCs were observed in the MS detection of another reaction sample after 50°C incubation (Figure S2, panel C and D). In a real-time measurement, small-molecule conjugating reagent is still active for lysine coupling, therefore the higher temperature could dramatically enhance the conjugation and lead to higher DARs. From these comparison data, we chose Endo-S as the ideal deglycosylation enzyme for real-time DAR measurement because of its exceeding activity under a mild condition.

### Real-time DAR monitoring for optimization of ADC lysine-conjugation condition

**Figure 4:**
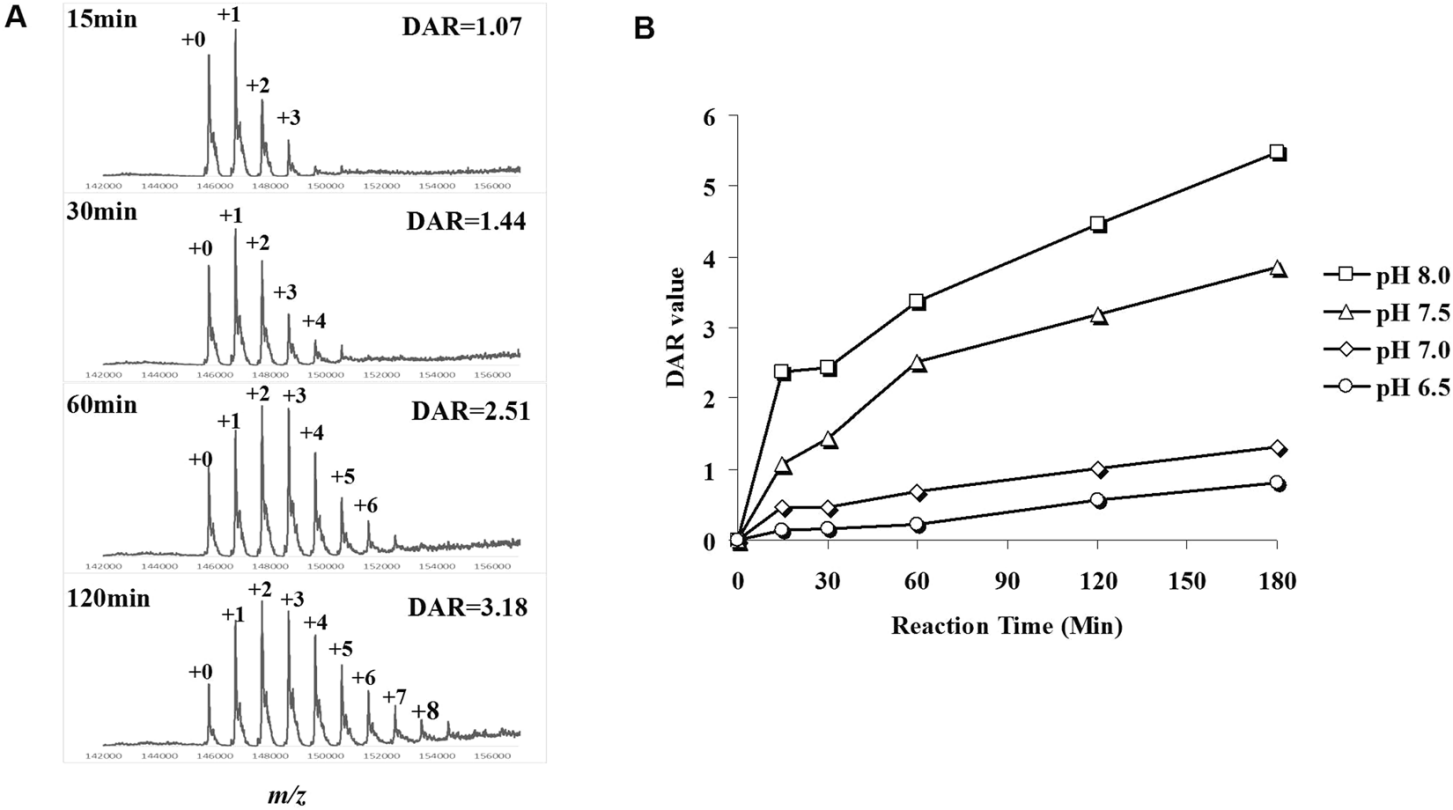
Real-time DAR detection of lysine-linked ADC for optimization of conjugation conditions. (**A**) representative LC-MS spectra of real-time DAR analysis on ADC **4** synthesized under pH 7.5; (**B**) real-time DAR analysis of ADC **4** synthesized under various pH values.

As described above, a 15 min procedure combined 5-min deglycosylation by Endo-S and 10 min LC-MS determination was developed for rapid analysis of DAR. Thereafter, we sought to apply this efficient approach for real-time monitoring on lysine-linked conjugation of ADC. A model reaction of DM1-SMCC with herceptin (transtuzumab) was set up for the monitoring. To optimize the conjugating condition, we performed the reaction under four different phosphate buffers (pH 6.5, 7.0, 7.5, and 8.0) respectively. At different time intervals, reaction aliquots were measured for DAR analysis. As shown in Fig. 4 panel A, a representative monitoring process was illustrated. After 15 min conjugation under the pH 7.5 buffer, the DAR of *in situ* ADC **4** was 1.07. As the conjugation proceeded, DAR increased to 1.44 at 30 min, 2.51 at 60 min, and 3.18 at 120 min. Time-course analysis of all four pH conditions was exhibited in Fig. 4 panel B and the detailed MS profiles were available in supplementary data (Figure S3). The conjugation was slow under pH 6.5 and 7.0, and the DAR only reached ~1 after 3 hrs. Under higher pH 7.5 and 8,0, the conjugation was dramatically enhanced and DAR increased to 4–5 within 2–3 hrs. On the basis of these real-time monitoring results, the optimal pH condition for lysine conjugation of ADC is about pH 7.5 which is mild for the antibody and the conjugation rate is moderate for quality control on DAR. We further validated the reproducibility of this real-time DAR measurement by testing three times for three different samples and the calculated DAR values were highly consistent (Figure S4). With the real-time monitoring, we employed the optimal condition and prepared the lysine-linked ADC **4** and **5** with expected DARs of ~3.5 (see supplementary data, Figure S5).

### Real-time DAR monitoring for dpADC (6) and gsADC (9)

**Figure 5:**
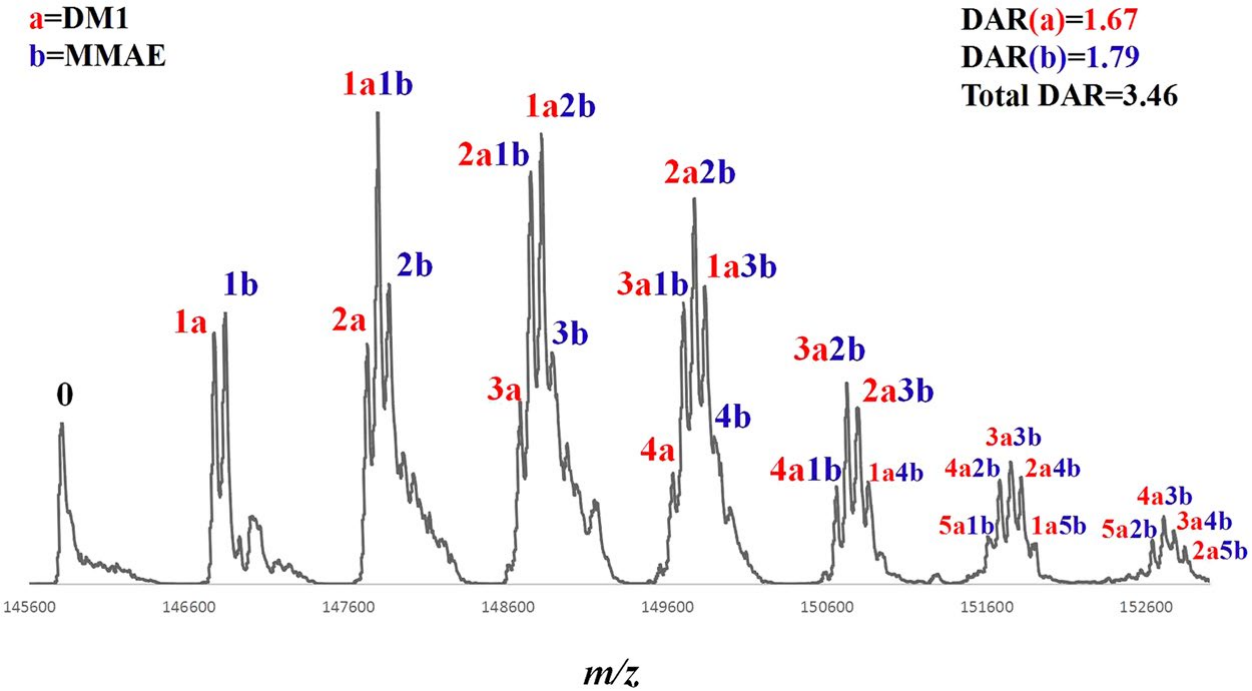
MS profile of dpADC **6** and DAR analysis (see detailed m/z assignment in supplementary data Table S1).

The successful real-time DAR analysis of lysine-linked ADC **4** demonstrated the efficacy of this approach. Next, we extended the application of this method in a more complicated case, the dual-payload ADC (dpADC) (**6**), which carries two different payloads with individual DAR values of each payload. It is more challenging for quality control of dpADC and there is an urgent need for rapid analytic technology on real-time monitoring. To control the total DAR of both payloads at ~3.5 (typical DAR value in clinical ADCs), we sought to control each payload with a DAR in the range of 1.5–2.0. Firstly, we introduced the drug-a (DM1) to herceptin under real-time monitoring until the DARa (DAR of drug-a) was ~1.6. Then, the ADC was purified through a protein-A affinity column, and the drug-b (MMAE) was added. The second-round conjugation was continuously monitored until the total DAR (DARa + DARb) reached the expected value (~3.5). Fig. 5 is the MS profile of the dpADC (**6**) with detailed DAR analysis. The deconvoluted MS peaks were assigned to corresponding ADC molecules with counted drug-a and drug-b numbers (marked on the top of individual peaks). The DARa and DARb were then calculated based on the MS intensity distribution. The deconvolution mass list was provided in supplementary data (Table S1).

**Figure 6:**
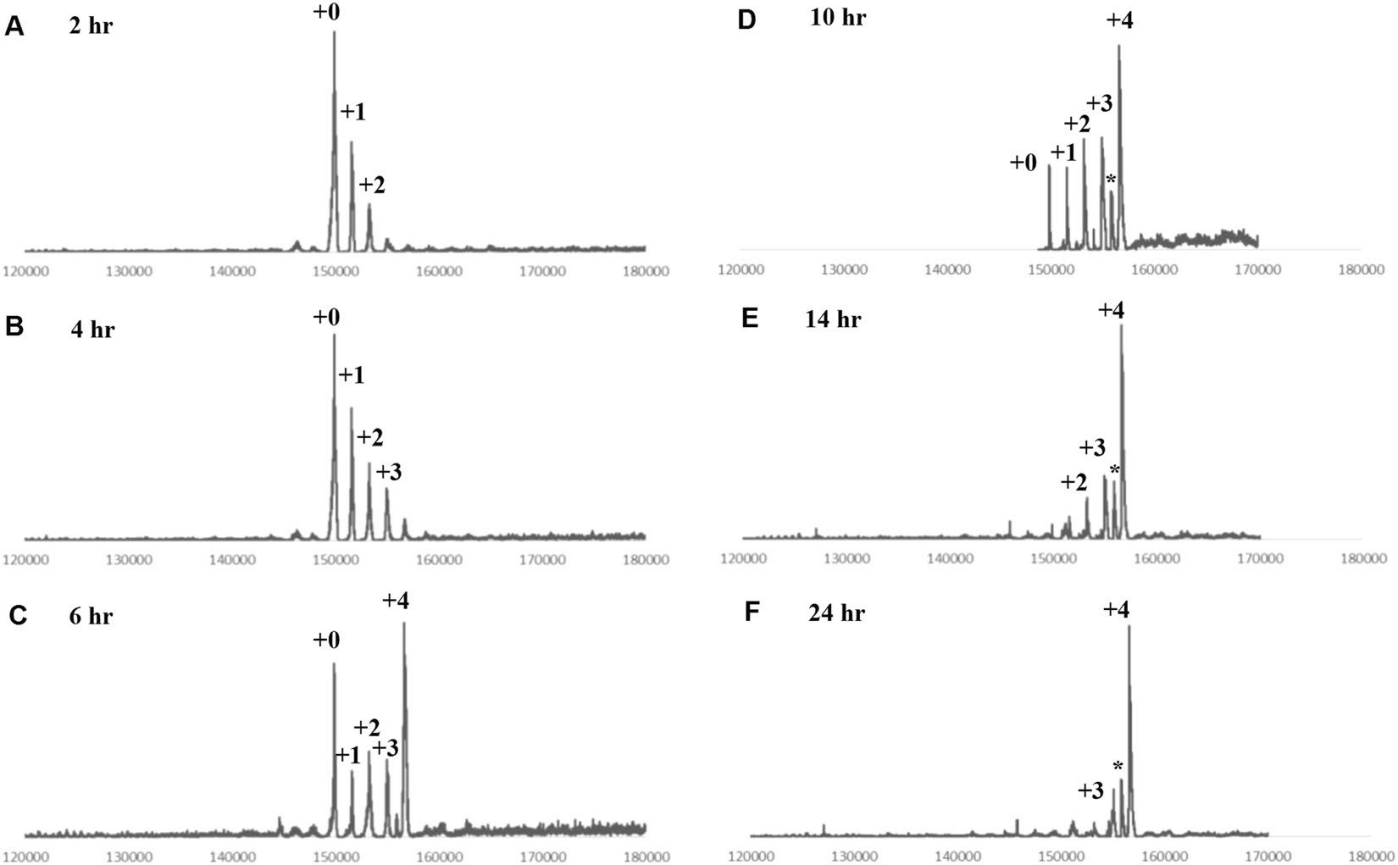
Real-time DAR analysis of gsADC **9**. The click reaction of DBCO-MMAE (**8**) with azido-herceptin (**7**) was monitored at (**A**) 2 hr; (**B**) 4 hr; (**C**) 6 hr; (**D**) 10 hr; (**E**) 14 hr; and (**F**) 24 hr.

Recently, we reported a new strategy of glycosite-specific ADC (gsADC) (**9**)^8^ as summarized in the Fig. 1. A major advantage of gsADC is the homogeneity of the glycan and conjugation site structures that is more convenient for the quality control. The real-time LC-MS determination could also be employed to monitor the chemoenzymatic glycoengineering and the click reaction. Although specific conjugating position and precise number (4) of the azido groups theoretically insured the fixed DAR of 4 in gsADC (**9**), it was still important to monitor the conjugation for condition optimization and method validation. In Fig. 6, the click reaction of the glycoengineered azido-herceptin (**7**) and DBCO-MMAE (**8**) was monitored directly by LC-MS without deglycosylation, because the payload was on the glycans and the homogeneous glycoform of **7** had already simplified the MS and DAR analysis. The DAR increased as the reaction time extended. After 24 hrs, the conjugation on total four azido groups was almost complete (Fig. 6, panel F). These monitoring data implicated that the click conjugation could be further optimized to shorten the reaction time, and the observed mass with partial decomposition (marked with asterisk in Fig. 6, panel D-F) suggested the stability of the payload linker need further improvement as well.

The above examples clearly demonstrated that the real-time LC-MS determination was a powerful tool in DAR analysis and control for various ADC subtypes including lysine-linked ADCs, dual-payload ADCs, and glycosite-specific ADCs.

### Real-time monitoring of IgG defucosylation

**Figure 7:**
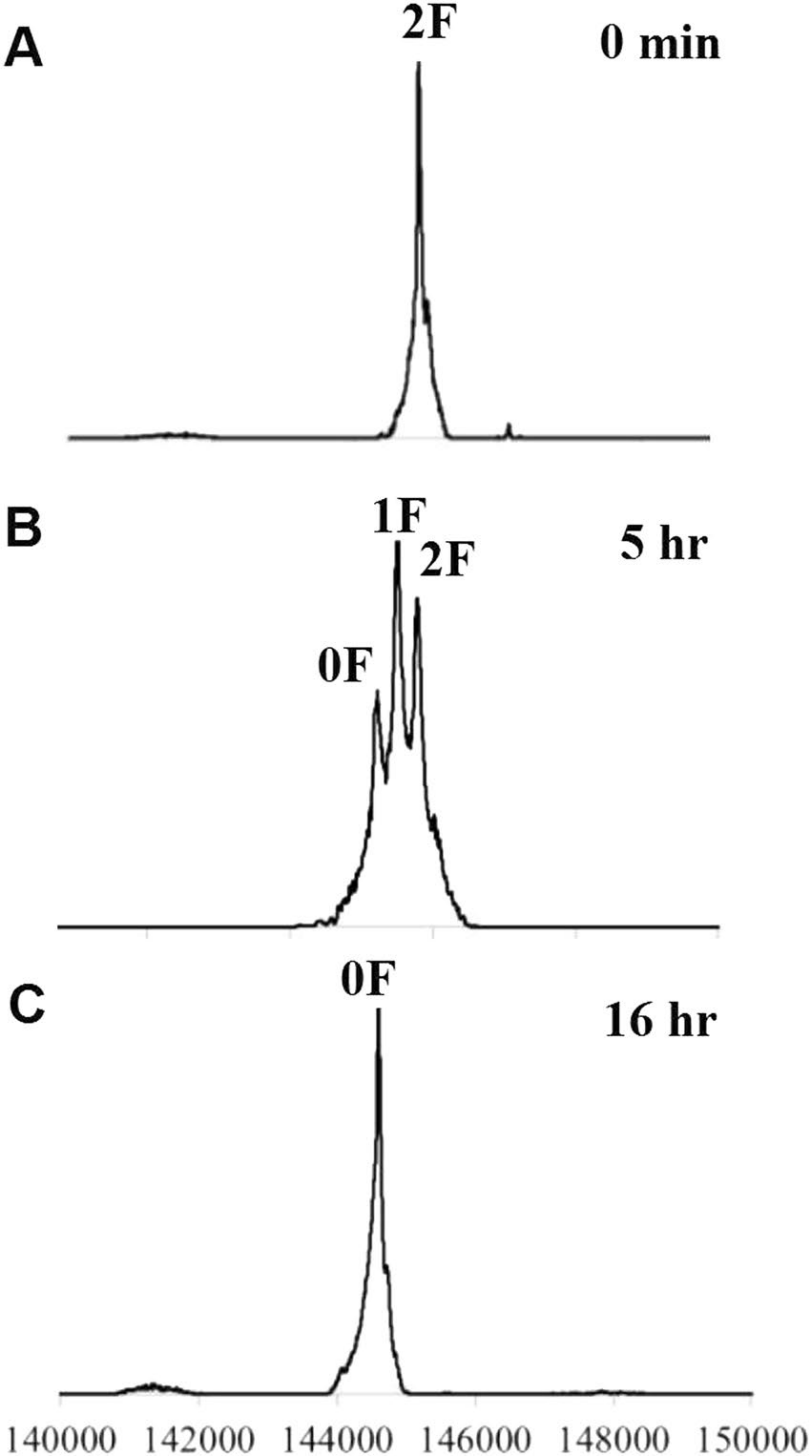
MS profiles of IgG defucosylation by a fucosidase under real-time monitoring. (**A**) IgG-Fucα1,6GlcNAc before treatment of fucosidase; (**B**) after treatment of fucosidase for 5 hours; (**C**) after treatment of fucosidase for 16 hours.

Besides the DAR detection, real-time LC-MS analysis of IgG heavy chain was also used in chemoenzymatic glycoengineering of antibodies^8, 38^. Here, we presented an example to exploit the real-time MS measurement of the intact IgG for monitoring of defucosylation. Fucose moieties on the native IgG Fc N-glycans hampers the binding of Fc domain with the FcγIIIa receptor^39^, therefore reduced the antibody-dependent cell-mediated cytotoxicity (ADCC) of therapeutic antibodies^40^. Defucosylation is a key procedure for glycoengineering of antibodies with better therapeutic efficacy^36, 41^. A fucosidase from *Lactobacillus casei* (AlfC)^42, 43^ was employed to hydrolyze the fucoses on the IgG-Fucα1,6GlcNAc which was obtained by Endo-S digestion (Figure S1). SDS-PAGE analysis was not able to monitor the defucosylation since the mass reduction (146 Da) is too small to detect by the band shift of the IgG heavy chain. The precise MS of intact IgG provides an excellent tool for deglycosylation monitoring. In Fig. 7 panel A, the IgG-Fucα1,6GlcNAc showed a homogeneous mass peak marked as 2F (containing 2 fucoses in both heavy chains). After treated with AlfC for 5 hrs (Fig. 7 panel B), the fucoses were partially removed as the MS profile showed the mixed glycoforms of 2F (2 fucoses), 1F (1 fucose), and 0F (no fucose). After 16 hrs, the defucosylation was complete and only 0F peak was observed.

## Conclusion

We reported here a real-time DAR analysis of ADCs via a procedure combined 5 min rapid deglycosylation by Endo-S and 10 min LC-MS determination. This approach was successfully applied in DAR monitoring of various ADC subtypes such as lysine-linked ADCs, dpADCs, and gsADCs. With the real-time detection, conjugation conditions such as the pH, temperature, reagent ratios, etc., could be easily optimized, and the quality control on DAR and ADC synthetic procedures was enabled as well. The complicated dual payloads with two DARs in dpADC could also be precisely controlled by this approach. In addition, real-time LC-MS detection of intact IgG also provided a perfect tool to monitor IgG modifications with small mass change such as defucosylation.

## Materials and Methods

### General

The enzymes of Endo-S, PNGase-F, and AlfC were expressed in *E. coli* following the reported procedures^8, 37, 42, 43^. The glycoengineered azido-herceptin (**7**) and DBCO-MMAE (**8**) were prepared following our previous paper^8^. DM1 (*N*_2_ *′*-deacetyl-*N*_2_ *′*-(3-mercapto-1-oxopropyl)- maytansine), MMAE (monomethyl auristain E), and SMCC (N-succinimidyl 4-(N-maleimidomethyl)cyclohexane-1-carboxylate) were purchased from Levena Biopharma (Nanjing, China). Other chemical reagents and solvents were purchased from Sinopharm Chemical Reagent Co. (Shanghai, China) or Sigma-Aldrich (Shanghai, China) and used without further purification. The MAbPac RP column (4 μm, 3.0 × 100mm) was purchased from ThermoFisher. Nuclear magnetic resonance (NMR) spectra were measured on a Varian-MERCURY Plus-400 or 500 instrument. ESI-HRMS spectra were measured on an Agilent 6230 LC-TOF MS spectrometer.

### High performance liquid chromatography (HPLC)

Analytical RP-HPLC was performed on a Beijing ChuangXinTongHeng LC3000 (analytic) instrument with a C18 column (5 μm, 4.6 × 150 mm) at 40 °C. The column was eluted with a linear gradient of 2–90% acetonitrile containing 0.1% TFA for 30 min at a flow rate of 1 mL min^−1^ (method A). Preparative HPLC was performed on a Beijing ChuangXinTongHeng LC3000 (preparative) instrument with a preparative column (Waters, C18, OBD, 5 μm, 19 × 250 mm) at room temperature. The column was eluted with a suitable gradient of aqueous acetonitrile containing 0.1% TFA at a flow rate of 10 mL min^−1^ (method B).

### Liquid chromatography mass spectrometry (LC-MS)

ESI-MS spectra of small molecules were measured on an Agilent 6230 LC–TOF MS spectrometer. The small molecules were analyzed using a short guard column and eluted with 70% methanol containing 0.1% formic acid. The mass spectra of small molecules were recorded in the mass range of 200–3000 or 600–2000 under a high resolution mass-spec mode (HRMS, standard 3200 m/z, 4 GHz). Key source parameters: a drying nitrogen gas flow of 11 L min−1; a nebulizer pressure of 40 psi; a gas temperature of 350 °C; a fragmenter voltage of 175 V; a skimmer voltage of 65 V; and a capillary voltage of 4000 V.

LC-MS spectra of antibodies and ADCs were measured on the same MS spectrometer (Agilent 6230) with a THERMO MAbPac RP column (4 μm, 3.0 × 100mm) at 80 °C. The column was eluted with an isocratic mobile phase of 20% acetonitrile (Buffer B) and 80% water containing 0.1% formic acid (Buffer A) for the first 3 min at a flow rate of 0.4 mL min^−1^, then it was successively eluted at the same flow rate with a linear gradient of 20–50% acetonitrile for additional 2.5 min, an isocratic 50% acetonitrile for 2 min, another linear gradient of 50–90% acetonitrile 0.5 min, and an isocratic 90% acetonitrile for 2 min. The mass spectra of antibodies were collected under the extended mass range mode (high 20 000 m/z, 1 GHz) in the mass range of 800–5000. Key source parameters: a drying nitrogen gas flow of 11 L min−1; a nebulizer pressure of 60 psi; a gas temperature of 350 °C; a fragmenter voltage of 400 V; a skimmer voltage of 65 V; and a capillary voltage of 5000 V. The multiple charged peaks of the antibody were deconvoluted using the Agilent MassHunter Bioconfirm software (deconvolution for protein, Agilent technology) with the deconvolution range from 100 kDa to 200 kDa; other parameters were set at default values for protein deconvolution. The TOF was calibrated over the range 0–5000 m/z using Agilent ESI calibration mix solution before analysis. The peak of MS 922 is the internal standard for calibration.

### Synthesis of DM1-SMCC (2)

DM1 (30.0 mg, 0.04 mmol, 1.0 eq) and N-succinimidyl 4-(N-maleimidomethyl)- cyclohexane-1-carboxylate (SMCC, 15.0 mg, 0.045 mmol, 1.1 eq) in a mixed solvent of acetonitrile and pH 7.5 phosphate buffer (v:v = 2:1) was stirred at r.t. for 2 hours under argon atmosphere. The residue was subject to semi-preparative HPLC for purification. The fractions containing the pure product were combined and lyophilized to get a white powder (40.7 mg, 95%). HRMS calcd. [M + H]^+^ 1072.3992, [M + Na]^+^ 1094.3811, found 1072.3974, 1094.3797. ^1^H NMR (400 MHz, Chloroform-d) δ 7.30 (d, J = 15.2 Hz, 1 H), 6.87 (dd, J = 5.2, 1.6 Hz, 1 H), 6.67 (dd, J = 5.3, 1.6 Hz, 1 H), 6.62 (d, J = 9.3 Hz, 1 H), 6.45 (dd, J = 15.3, 11.1 Hz, 1 H), 5.64 (dd, J = 15.1, 9.0 Hz, 1 H), 5.35 (m, 1 H), 4.76 (dt, J = 12 Hz, 1 H), 4.36 (t, J = 11.4 Hz, 1 H), 4.10 (s, 3 H), 3.75 (ddd, J = 20.2, 9.1, 3.5 Hz, 1 H), 3.67 (dd, J = 12.7, 5.2 Hz, 1 H), 3.53 (d, J = 9.0 Hz, 1 H), 3.39 (d, J = 3.3 Hz, 3 H), 3.37 (s, 1 H), 3.33–2.94 (m, 11 H), 2.90 (s, 3 H), 2.86 (s, 4 H), 2.77-2.52 (m, 3 H), 2.41 (ddd, J = 18.8, 9.3, 3.5 Hz, 1 H), 2.33-2.22 (dd, J = 14.4, 2.8 Hz, 1 H), 2.16 (d, J = 13.3 Hz, 2 H), 1.86-1.42 (m, 10 H), 1.35 (d, J = 6.8 Hz, 3 H), 1.30 (d, J = 6.4 Hz, 4 H), 1.07 (m, 2 H), 0.81 (s, 3 H). ^13^C NMR (125 MHz, CDCl3) δ 176.95, 176.85, 174.70, 171.01, 170.81, 170.53, 169.28, 169.24, 169.18, 156.02, 154.26, 154.20, 141.91, 141.08, 139.48, 133.33, 127.46, 125.17, 121.96, 118.70, 115.89, 113.23, 88.11, 80.80, 78.01, 77.28, 77.02, 76.77, 74.69, 66.99, 59.89, 56.71, 56.61, 46.61, 44.43, 44.36, 40.32, 39.58, 39.49, 38.81, 35.71, 35.66, 35.59, 35.41, 35.32, 32.44, 29.30, 29.22, 28.00, 27.11, 25.59, 15.53, 14.45, 13.44, 13.40, 12.11.

### Synthesis of MMAE-SMCC (3)

3-Mercaptopropanoic acid (1.0 g, 9.4 mmol) was dissolved in water (30 mL) and was cooled to 0 °C with an ice bath. Methyl methanethiolsulfonate (1.31 g, 10.4 mmol) in absolute ethanol (15 mL) was then added to the solution. The mixture was stirred at r.t. overnight, then, the residue was diluted with saturated brine (80 mL) and extracted with ether. The combined organic layers were then washed with saturated brine and dried over anhydrous Na_2_SO_4_. After filtration, the solvent was removed to afford the product 3-(methyldithio)propanoic acid as a colorless oil. The crude product was directly used in next step without further purification.

To a stirring solution of 3-mercaptopropanoic acid (200 mg, 1.32 mmol) in dichloromethane (5 mL), N-hydroxysuccinimide (227 mg, 1.98 mmol) and 1-[3-(dimethylamino)propyl]-3- thylcarbodiimide hydrochloride (EDC, 380 mg, 1.98 mmol) were added. The mixture was stirred at r.t. under an argon atmosphere for 2 h. The residue was diluted with ethyl acetate (40 mL) and washed with 50 mM potassium phosphate buffer at pH 6.0 (2 × 20 mL) then saturated sodium chloride (20 mL). The organic layer was dried over anhydrous Na_2_SO_4_ and filtered. The solvent was removed under vacuum, and the resulted solid was purified by column chromatography to give the product 2, 5-dioxopyrrolidin-1-yl 3-(methylsulfinothioyl)propanoate as a white powder (172 mg, 52.3%). ^1^H NMR (400 MHz, Chloroform-d) δ 3.11 (ddd, J = 7.7, 6.5, 1.6 Hz, 2 H), 3.03 (ddd, J = 8.5, 6.5, 1.6 Hz, 2 H), 2.92 − 2.80 (m, 4 H), 2.46 (s, 3 H). ^13^C NMR (100 MHz, Chloroform-d) δ 168.93, 167.14, 31.35, 31.19, 25.59, 23.20.

To a solution of MMAE (30 mg, 0.042 mmol) in acetonitrile (5 mL), 2,5-dioxopyrrolidin-1-yl 3-(methylsulfinothioyl)propanoate (52.3 mg, 0.21 mmol) was added. Then, 50 mM sodium phosphate buffer at pH = 7.5 (2.5 mL) was added in the reaction. The mixture was stirred at r.t. for 48 h, then was subject to semi-preparation HPLC purification. The product of 3-(methyldithio)propanoic MMAE was obtained as a white power (14.3 mg, 40.2%). HRMS. Calcd for [M + H] + 852.4979, [M + Na] + 874.4798; found 852.4940, 874.4762.

To a solution of 3-(methyldithio)propanoic MMAE (10 g, 0.0124 mmol) in acetonitrile (2 mL), Tris(2-carboxyethyl)phosphine hydrochloride (TCEP, 7.1 mg, 0.0248 mmol) in a neutral aqueous solution was added. The reaction was stirred at room temperature for 2 h, then was subject to semi-preparation HPLC purification. The product of 3-mercaptopropanoic MMAE was obtained as a white power (9.1 mg, 95%). HRMS. Calcd for [M + H] + 806.5102, [M + Na] + 828.4921; found 806.5143, 828.4964.

To a solution of 3-mercaptopropanoic MMAE (5 mg, 0.0062 mmol) in a mixture of acetonitrile (2 mL) and 50 mM sodium phosphate buffer at pH = 7.5 (1 mL), N-Succinimidyl 4-(N-maleimidomethyl)cyclohexane-1-carboxylate (SMCC, 2.27 mg, 0.0068 mmol) was added under argon at room temperature. After 1 h, the residue was subject to semi-preparation HPLC purification. The product of MMAE-SMCC (**3**) was obtained as a white power (6.6 mg, 95%). HRMS. Calcd for [M + H] + 1140.6266, [M + Na] + 1162.6086; found 1140.6211, 1162.6037. ^1^H NMR (500 MHz, Chloroform-d) δ 7.43 − 7.32 (m, 4 H), 6.62 (d, J = 6.1 Hz, 1 H), 4.96 (d, J = 3.0 Hz, 1 H), 4.77 − 4.57 (m, 3 H), 4.36 (s, 1 H), 4.28 (tt, J = 8.3, 4.1 Hz, 2 H), 4.17 (d, J = 7.0 Hz, 1 H), 4.06 (s, 1 H), 3.91 − 3.75 (m, 2 H), 3.55 (s, 1 H), 3.48 − 3.37 (m, 7 H), 3.35 (s, 1 H), 3.32 (s, 3 H), 3.31 − 3.20 (m, 1 H), 3.20 − 3.08 (m, 3 H), 3.08 − 2.89 (m, 7 H), 2.83 (t, J = 3.8 Hz, 5 H), 2.80 − 2.68 (m, 2 H), 2.66 − 2.44 (m, 4 H), 2.44 − 2.34 (m, 2 H), 2.21 − 2.15 (m, 3 H), 2.06 (dddd, J = 34.3, 20.4, 10.5, 5.6 Hz, 4 H), 1.91 − 1.83 (m, 2 H), 1.56 (q, J = 13.2 Hz, 3 H), 1.38 (s, 1 H), 1.27 (dd, J = 7.1, 2.5 Hz, 4 H), 1.15 − 1.01 (m, 7 H), 0.99 (d, J = 6.6 Hz, 4 H), 0.96 − 0.91 (m, 6 H), 0.89 (d, J = 8.3 Hz, 3 H), 0.88 − 0.74 (m, 11 H). ^13^C NMR (125 MHz, Chloroform-d) δ 174.30, 172.41, 171.34, 170.25, 170.05, 169.26, 168.64, 127.76, 127.56, 126.81, 125.82, 125.70, 81.40, 78.11, 75.29, 62.09, 60.47, 59.63, 57.49, 53.57, 51.14, 47.38, 44.42, 43.91, 39.86, 39.36, 39.22, 37.13, 35.44, 35.37, 34.87, 33.61, 32.84, 30.47, 30.16, 28.81, 28.74, 27.51, 27.02, 26.81, 25.58, 25.23, 25.10, 24.49, 24.40, 18.81, 18.14, 17.45, 15.46, 13.97, 13.43, 10.38.

### General procedures for synthesis of lysine-linked ADCs, dp ADCs, and gsADCs

For lysine-linked ADC: A solution of herceptin (1 mg/mL) and the SMCC-linked small drug (**2** or **3**, 10–12 eq.) in a phosphate buffer (pH 7.5, 50 mM) containing 4–5% DMSO was incubated at 25 °C. The conjugation reaction was monitored by LC-MS after deglycosylation. Until the DAR reached the target value, the reaction mixture was immediately subject to a pre-prepared protein-A affinity column for purification. Before loading the ADC sample, the protein A-agarose column was pre-washed with a glycine-HCl (100 mM, pH2.5, 5 column volume) and pre-equilibrated with PB (50 mM, pH 8.0, 5 column volume). After loading the ADC, the column was washed with PB (50 mM, pH8.0, 5 column volume) and glycine-HCl (20 mM, pH5.0, 3 column volume) successively and the bound ADC was eluted with glycine-HCl (100 mM, pH2.5, 5 column volume) followed by neutralization to ~ pH 7.5 with glycine-HCl (1 M, pH 8.8) immediately. The fractions contained the target ADCs were combined and concentrated by centrifugal filtration through a 10 kDa cut-off membrane. And the concentration of the product was measured by Bicinchoninic Acid (BCA) Kit for Protein Determination following the manufacture’s protocol.

ADC (**4**). DAR 3.46; LC-MS deconvolution data: 145805.17 (+0 DM1), 146775.09 (+1 DM1), 147720.05 (+2 DM1), 148681.07 (+3 DM1), 149635.76 (+4 DM1), 150601.21 (+5 DM1), 151554.03 (+6 DM1).

ADC (**5**). DAR 3.53; LC-MS deconvolution data: 145809.88 (+0 MMAE), 146839.36 (+1 MMAE), 147867.22 (+2 MMAE), 148891.49 (+3 MMAE), 149917.97 (+4 MMAE), 150943.92 (+5 MMAE), 151968.39 (+6 MMAE).

For dual-payload ADC (**6**): A solution of herceptin (1 mg/mL) and the DM1-SMCC (**2**, 6 eq.) in a phosphate buffer (pH 7.5, 50 mM) containing 5% DMSO was incubated at 25 °C. The conjugation reaction was monitored by LC-MS after deglycosylation. After 45 min, the DAR(DM1) was 1.67 then the mixture was immediately subject to a protein-A affinity column for purification. The resulted DM1-ADC (1 mg/mL) was treated with MMAE-SMCC (**3**, 5 eq.) in a phosphate buffer (pH 7.5, 50 mM) containing 5% DMSO was incubated at 25 °C. The reaction was monitored until the total DAR (DM1 + MMAE) reached ~3.5. The residue was then immediately subject to a protein-A affinity column for purification. The detailed MS list was shown in Table S1.

For glycosite-specific ADC (**9**): The azido Herceptin (**7**) (1 mg/mL) was incubated with DBCO-MMAE (**8**) (20 eq.) in a phosphate butter (50 mM, pH 7.5) containing 10% DMSO at 30 °C and monitored by LC-MS. After the conjugation, the reaction mixture was subject to affinity chromatography via protein A resin following above procedure. Fractions containing the products were combined to give ADC **9**. LC-MS deconvolution data: 156659.32.

### Deglycosylation of ADCs with Endo-S and PNGase-F

For Endo-S: A 20 μL aliquot of *in situ* ADCs (1 mg/mL) was taken from the reaction solution and treated with Endo-S (1.5 μg/mL). The mixture was incubated at 25 °C for 5 min then was subject to SDS-PAGE analysis and LC-MS determination.

For PNGase-F: The ADC **4** was purified through a protein-A affinity column and the purified sample (1 mg/mL) was treated with PNGase-F (200 μg/mL). The mixture was incubated at 37 °C for 12 hr then was subject to SDS-PAGE analysis and LC-MS determination.

### pH optimization by real-time LC-MS monitoring and DAR analysis of ADC **4**

A solution of herceptin (1 mg/mL) and DM1-SMCC (**2**, 12 eq.) in a phosphate buffer (50 mM, pH 6.5, 7.0, 7.5, or 8.0) containing 5% DMSO was incubated at 25 °C. At interval time of 15, 30, 60, 120, and 180 min, 20 μL reaction aliquots from each pH vial was taken out and treated with Endo-S (1.5 μg/mL) at 25 °C for 5 min. Then the mixture was subject to LC-MS determination and DAR analysis. The time-course results were presented in Figs 4 and S3.

### DAR quality control of ADC **4-6** by real-time monitoring

The above reaction solution of herceptin and SMCC-linked small molecules was monitored following the Endo-S deglycosylation and LC-MS determination methods descript above for every 15 min. Once the DAR value reached the target (~3.5), the reaction was stopped and subject to the protein-A affinity column immediately. The purified ADCs **4–6** with controlled DARs were validated with additional LC-MS measurement (Figure S5).

### IgG defucosylation with AlfC and real-time LC-MS monitoring

Rituximab (20 mg/mL) in a Tris-Cl buffer (50 mM, pH 7.5) was treated with Endo-S (15 μg/mL) for 5 min, then AlfC (1 mg/mL) was added and the mixture was incubated at 37 °C. The aliquots were taken from the reaction and subject to LC-MS determination directly. LC-MS deconvolution data: 144894.20 (2 F, containing two fucoses), 144749.13 (1 F, containing one fucose), 144602.67 (0 F, no fucose).

## Electronic supplementary material

Supplementary Information

## References

[1] Donaghy H (2016). Effects of antibody, drug and linker on the preclinical and clinical toxicities of antibody-drug conjugates. MAbs.

[2] Jain N, Smith SW, Ghone S, Tomczuk B (2015). Current ADC Linker Chemistry. Pharm. Res..

[3] Sassoon I, Blanc V (2013). Antibody-drug conjugate (ADC) clinical pipeline: a review. Methods Mol. Biol..

[4] Carter PJ, Senter PD (2008). Antibody-drug conjugates for cancer therapy. Cancer J..

[5] Drake PM, Rabuka D (2015). An emerging playbook for antibody-drug conjugates: lessons from the laboratory and clinic suggest a strategy for improving efficacy and safety. Curr. Opin. Chem. Biol..

[6] Shinmi D (2016). One-Step Conjugation Method for Site-Specific Antibody-Drug Conjugates through Reactive Cysteine-Engineered Antibodies. Bioconjug. Chem..

[7] Zhou Q, Kim J (2015). Advances in the Development of Site-Specific Antibody-Drug Conjugation. Anticancer Agents Med. Chem..

[8] Feng T (2016). One-pot N-glycosylation remodeling of IgG with non-natural sialylglycopeptides enables glycosite-specific and dual-payload antibody-drug conjugates. Org. Biomol. Chem..

[9] van Geel R (2015). Chemoenzymatic Conjugation of Toxic Payloads to the Globally Conserved N-Glycan of Native mAbs Provides Homogeneous and Highly Efficacious Antibody-Drug Conjugates. Bioconjug. Chem..

[10] Tian F (2014). A general approach to site-specific antibody drug conjugates. Proc. Natl. Acad. Sci. USA.

[11] Zhu Z (2014). Site-specific antibody-drug conjugation through an engineered glycotransferase and a chemically reactive sugar. MAbs.

[12] Li X, Fang T, Boons GJ (2014). Preparation of well-defined antibody-drug conjugates through glycan remodeling and strain-promoted azide-alkyne cycloadditions. Angew. Chem. Int. Ed..

[13] Behrens CR, Liu B (2014). Methods for site-specific drug conjugation to antibodies. MAbs.

[14] Axup JY (2012). Synthesis of site-specific antibody-drug conjugates using unnatural amino acids. Proc. Natl. Acad. Sci. USA.

[15] Bhakta S, Raab H, Junutula JR (2013). Engineering THIOMABs for site-specific conjugation of thiol-reactive linkers. Methods Mol. Biol..

[16] Burke PJ (2017). Optimization of a PEGylated Glucuronide-Monomethylauristatin E Linker for Antibody-Drug Conjugates. Mol. Can. Ther..

[17] Staben LR (2016). Targeted drug delivery through the traceless release of tertiary and heteroaryl amines from antibody-drug conjugates. Nat. Chem..

[18] Lyon RP (2015). Reducing hydrophobicity of homogeneous antibody-drug conjugates improves pharmacokinetics and therapeutic index. Nat. Biotechnol..

[19] Puthenveetil S (2016). Natural Product Splicing Inhibitors: A New Class of Antibody-Drug Conjugate (ADC) Payloads. Bioconjug. Chem..

[20] Levengood MR (2017). Orthogonal Cysteine Protection Enables Homogeneous Multi-Drug Antibody-Drug Conjugates. Angew. Chem. Int. Ed..

[21] Bakhtiar R (2016). Antibody drug conjugates. Biotechnol. Lett..

[22] Hamblett KJ (2004). Effects of drug loading on the antitumor activity of a monoclonal antibody drug conjugate. Clin. Can. Res..

[23] Wakankar A, Chen Y, Gokarn Y, Jacobson FS (2011). Analytical methods for physicochemical characterization of antibody drug conjugates. MAbs.

[24] Stump B, Steinmann J (2013). Conjugation process development and scale-up. Methods Mol. Biol..

[25] Chen Y (2013). Drug-to-antibody ratio (DAR) by UV/Vis spectroscopy. Methods Mol. Biol..

[26] Bobaly B, Randazzo GM, Rudaz S, Guillarme D, Fekete S (2016). Optimization of non-linear gradient in hydrophobic interaction chromatography for the analytical characterization of antibody-drug conjugates. J. Chromatogr. A.

[27] Ouyang J (2013). Drug-to-antibody ratio (DAR) and drug load distribution by hydrophobic interaction chromatography and reversed phase high-performance liquid chromatography. Methods Mol. Biol..

[28] Huang RY, Chen G (2016). Characterization of antibody-drug conjugates by mass spectrometry: advances and future trends. Drug Discov. Today.

[29] Basa L (2013). Drug-to-antibody ratio (DAR) and drug load distribution by LC-ESI-MS. Methods Mol. Biol..

[30] Wagner-Rousset E (2015). Antibody-drug conjugate model fast characterization by LC-MS following IdeS proteolytic digestion. MAbs.

[31] Xu K (2011). Characterization of intact antibody-drug conjugates from plasma/serum *in vivo* by affinity capture capillary liquid chromatography-mass spectrometry. Anal. Biochem..

[32] Debaene F (2014). Innovative native MS methodologies for antibody drug conjugate characterization: High resolution native MS and IM-MS for average DAR and DAR distribution assessment. Anal. Chem..

[33] Redman EA, Mellors JS, Starkey JA, Ramsey JM (2016). Characterization of Intact Antibody Drug Conjugate Variants Using Microfluidic Capillary Electrophoresis-Mass Spectrometry. Anal. Chem..

[34] Widdison WC (2006). Semisynthetic maytansine analogues for the targeted treatment of cancer. J. Med. Chem..

[35] Baskin JM (2007). Copper-free click chemistry for dynamic *in vivo* imaging. Proc. Natl. Acad. Sci. USA.

[36] Huang W, Giddens J, Fan SQ, Toonstra C, Wang LX (2012). Chemoenzymatic glycoengineering of intact IgG antibodies for gain of functions. J. Am. Chem. Soc..

[37] Sjogren J (2015). EndoS and EndoS2 hydrolyze Fc-glycans on therapeutic antibodies with different glycoform selectivity and can be used for rapid quantification of high-mannose glycans. Glycobiology.

[38] Parsons TB (2016). Optimal Synthetic Glycosylation of a Therapeutic Antibody. Angew. Chem. Int. Ed..

[39] Ferrara C (2011). Unique carbohydrate-carbohydrate interactions are required for high affinity binding between FcgammaRIII and antibodies lacking core fucose. Proc. Natl. Acad. Sci. USA.

[40] DiLillo DJ, Ravetch JV (2015). Fc-Receptor Interactions Regulate Both Cytotoxic and Immunomodulatory Therapeutic Antibody Effector Functions. Cancer Immunol. Res..

[41] Beck A, Reichert JM (2012). Marketing approval of mogamulizumab: a triumph for glyco-engineering. MAbs.

[42] Rodriguez-Diaz J, Monedero V, Yebra MJ (2011). Utilization of natural fucosylated oligosaccharides by three novel alpha-L-fucosidases from a probiotic Lactobacillus casei strain. Appl. Environ. Microbiol..

[43] Li TZ, Tong X, Yang Q, Giddens JP, Wang LX (2016). Glycosynthase Mutants of Endoglycosidase S2 Show Potent Transglycosylation Activity and Remarkably Relaxed Substrate Specificity for Antibody Glycosylation Remodeling. J. Biol. Chem..

