# Supplementary material for: Real-Time Analysis on Drug-Antibody Ratio of Antibody-Drug Conjugates for Synthesis, Process Optimization, and Quality Control
Source: Sci Rep. 2017 Aug 10;7:7763. doi: 10.1038/s41598-017-08151-2 (PMC5552727; doi:10.1038/s41598-017-08151-2)
Supplement: Supplementary file 1 — Supplementary Information [file 41598_2017_8151_MOESM1_ESM.pdf]

Supplementary data for:

Real-Time Analysis on Drug-Antibody Ratio of Antibody-Drug Conjugates for Synthesis Process Optimization and Quality Control

Yubo Tang[1,2], Feng Tang[2], Yang Yang[2], Lei Zhao[2], Hu Zhou[2], Jinhua Dong[1,*], Wei Huang[2,*]

[1]Key Laboratory of Structure-Based Drug Design and Discovery, Ministry of Education, Shenyang Pharmaceutical University, Shenyang, 110016, China

[2]CAS Key Laboratory of Receptor Research, CAS Center for Excellence in Molecular Cell Science, Shanghai Institute of Materia Medica, Chinese Academy of Sciences, 555 Zuchongzhi Road, Pudong, Shanghai, China 201203

Scheme S1. Synthesis of DM1-SMCC (**2**)

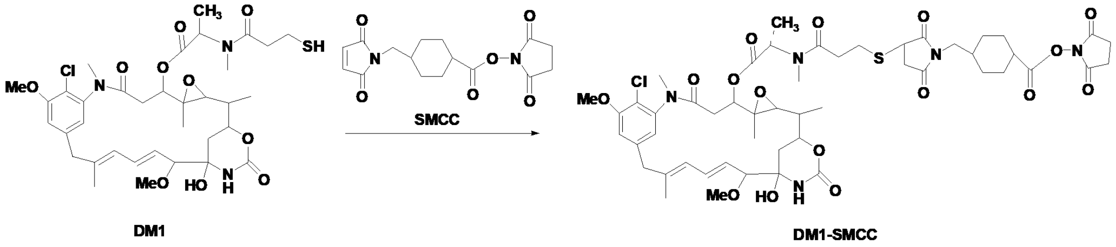

Scheme S2. Synthesis of MMAE-SMCC (**3**)

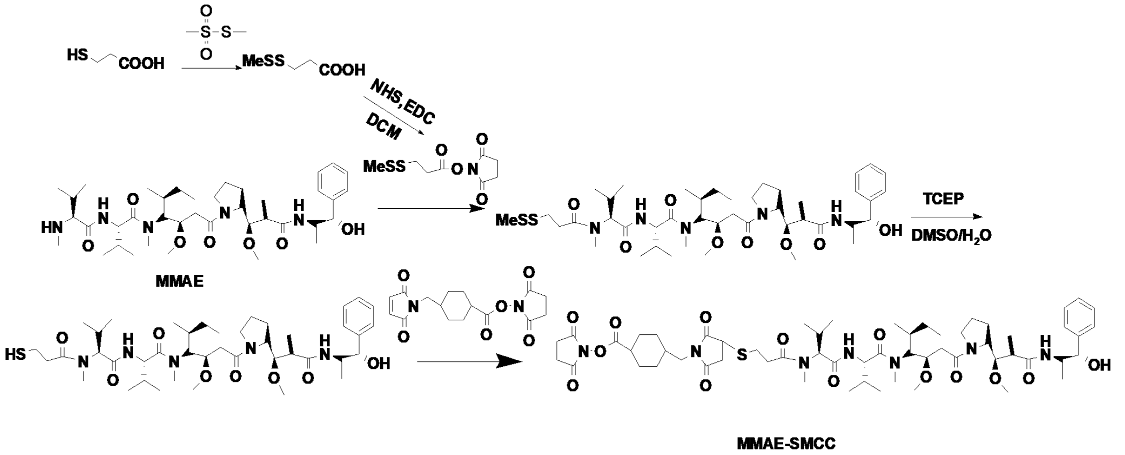

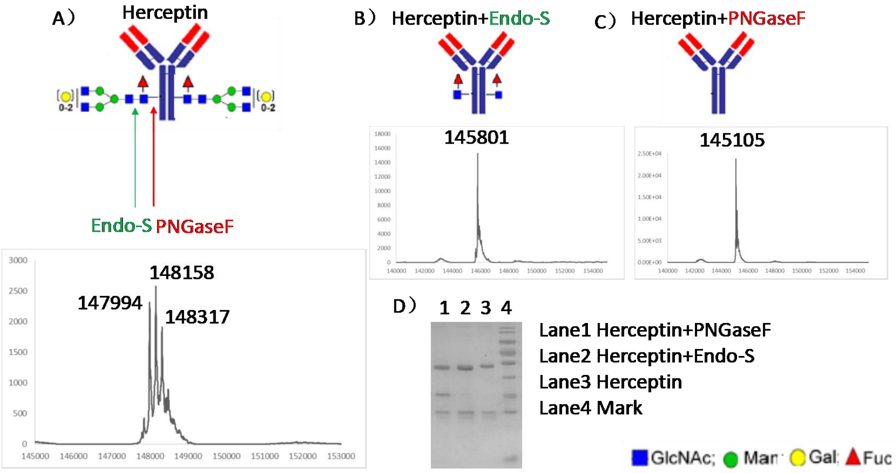

Figure S1. Deglycosylation of IgG with PNGase-F and Endo-S. A) upper: hydrolytic sites of Endo-S and PNGase-F; bottom: deconvolution MS profile of native herceptin containing mixed glycoforms of G0F, G1F, and G2F in both heavy chains; B) MS profile of deglycosylated herceptin with Endo-S; C) MS profile of deglycosylated herceptin with PNGase-F; D) SDS-PAGE analysis of herceptin deglycosylation with Endo-S and PNGase-F.

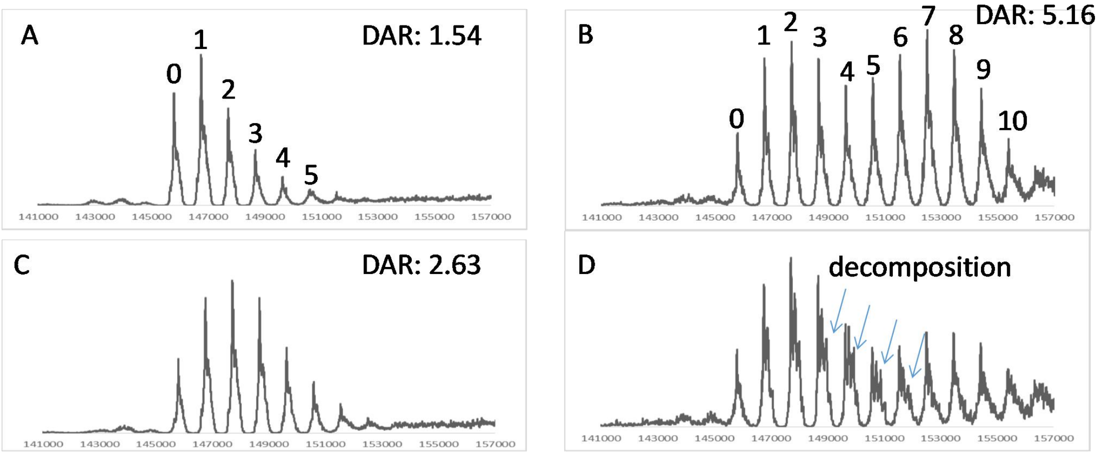

Figure S2. Deglycosylation temperature affects DAR measurement. Panel A: LC-MS profile and DAR value of *in situ* ADC **4** (pH 7.5, 30 min) after deglycosylation with Endo-S at 25 ^{o}C for 10 min; Panel B: LC-MS profile and DAR value of *in situ* ADC **4** (pH 7.5, 30 min) after deglycosylation with Endo-S at 50 ^{o}C for 10 min; Panel C: LC-MS profile and DAR value of *in situ* ADC **4** (pH 7.5, 60 min) after deglycosylation with Endo-S at 25 ^{o}C for 10 min; Panel D: LC-MS profile of *in situ* ADC **4** (pH 7.5, 60 min) after deglycosylation with Endo-S at 50 ^{o}C for 10 min. Decomposition of ADC **4** was observed and marked with arrows.

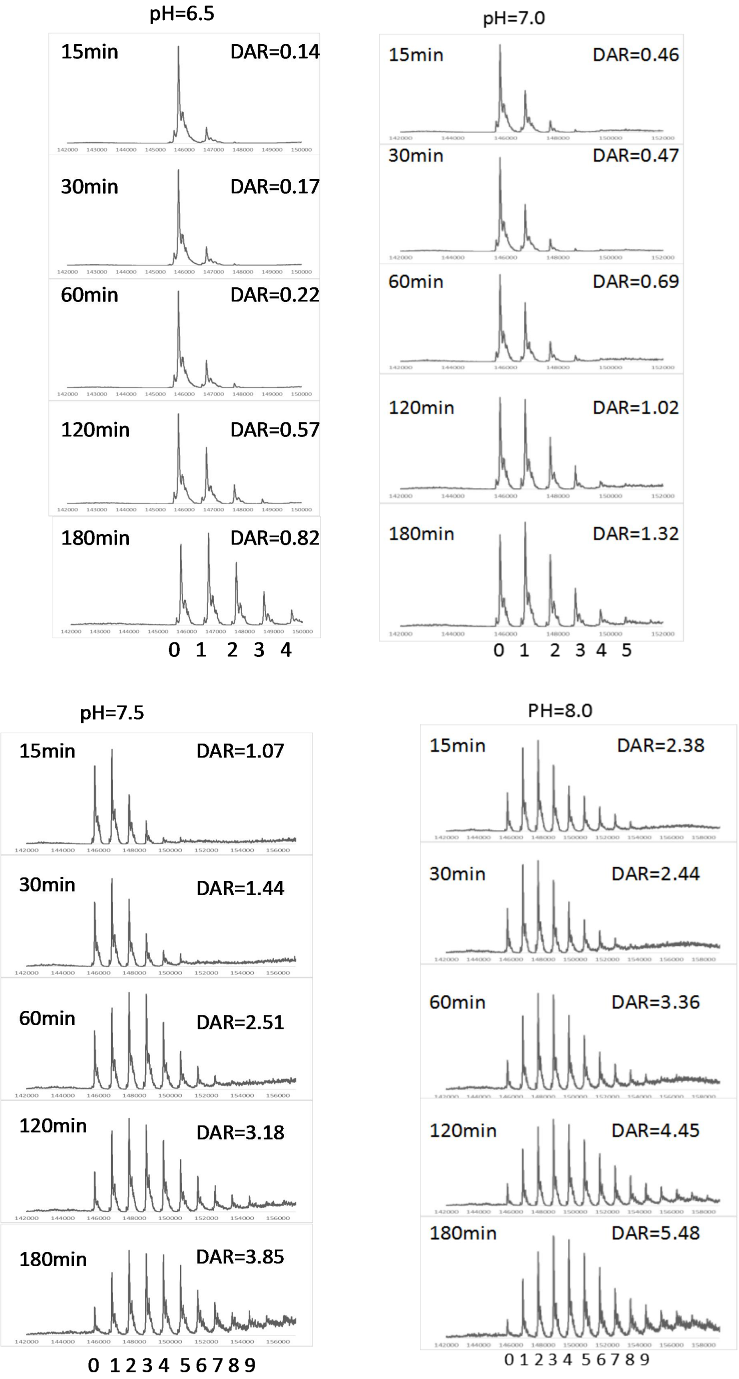

Figure S3. Real-time LC-MS determination and DAR analysis of *in situ* ADC (**4**) under four different pH conditions.

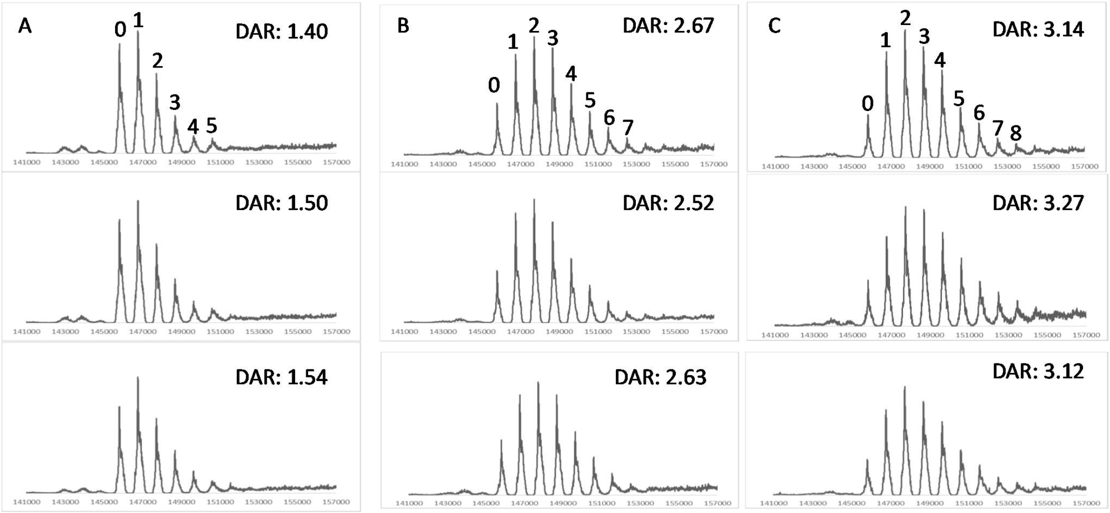

Figure S4. Method validation by repeat DAR measurements. Panel A: Triplicate DAR measurements of *in situ* ADC **4** (pH 7.5, 30 min); Panel B: Triplicate DAR measurements of *in situ* ADC **4** (pH 7.5, 60 min); Panel C: Triplicate DAR measurements of *in situ* ADC **4** (pH 7.5, 120 min).

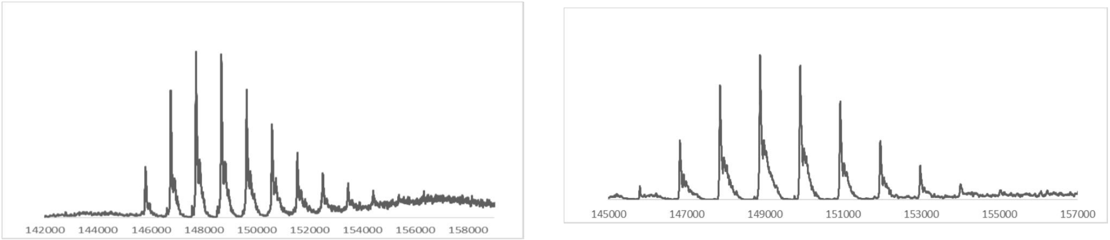

T-DM1 (4) DAR=3.46 **T-MMAE (5) DAR=3.53**

Figure S5. MS profiles of ADC **4** and **5** with controlled DAR values.

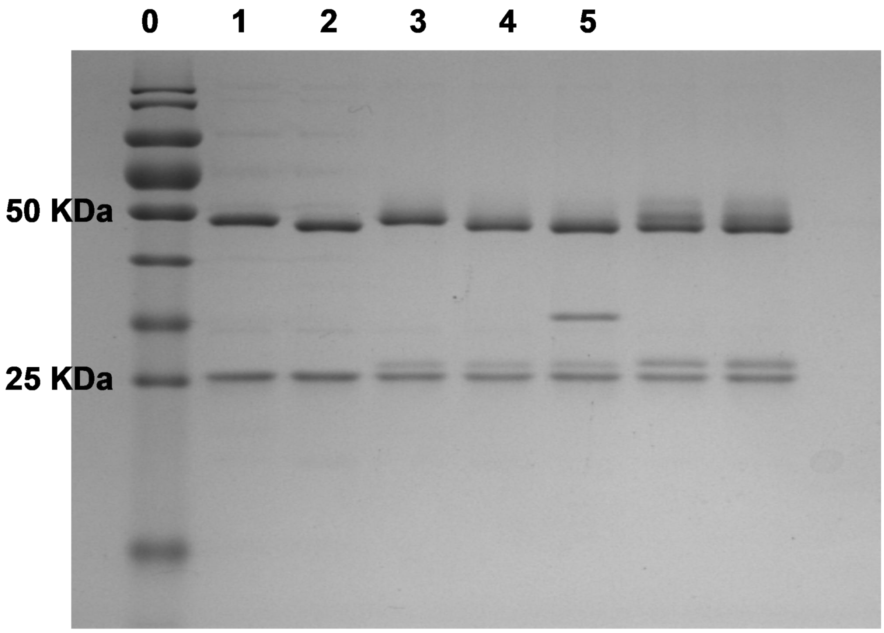

Figure S6. Original full-size SDS-PAGE of Figure 2B in the paper.

Table S1. Deconvolution mass list of dual-payload ADC (**6**). Drug-A: DM1, Drug-B: MMAE

Compound	Calculated mass (Dalton)	Found mass (Dalton)	Error （Dalton）	Error （ppm）
0	145806.06	145809.49	3.43	23.52
1A	146763.43	146764.15	0.72	4.91
1B	146831.66	146829.81	-1.85	12.60
2A	147720.80	147720.23	-0.57	3.86
1A1B	147789.03	147789.52	0.49	3.32
2B	147857.26	147855.00	-2.26	15.29
3A	148678.17	148679.54	1.37	9.21
2A1B	148746.40	148747.06	0.66	4.44
1A2B	148814.63	148815.04	0.41	2.76
3B	148882.86	148880.62	-2.24	15.05
4A	149635.54	149634.27	-1.27	8.49
3A1B	149703.77	149703.73	-0.04	0.27
2A2B	149772.00	149771.31	-0.69	4.61
1A3B	149840.23	149839.99	-0.24	1.60
4B	149908.46	149909.23	0.77	5.14
4A1B	150661.14	150660.17	-0.97	6.44
3A2B	150729.37	150728.94	-0.43	2.85
2A3B	150797.60	150797.52	-0.08	0.53
1A4B	150865.83	150864.44	-1.39	9.21
5B	150934.06	150936.26	2.20	14.58
5A1B	151618.51	151617.50	-1.01	6.66
4A2B	151686.74	151686.45	-0.29	1.91
3A3B	151754.97	151756.75	1.78	11.73
2A4B	151823.20	151819.08	-4.12	27.14
1A5B	151891.43	151888.65	-2.78	18.30
5A2B	152644.11	152646.12	2.01	13.17
4A3B	152712.34	152718.68	6.34	41.52
3A4B	152780.57	152778.35	-2.22	14.53
2A5B	152848.80	152844.91	-3.89	25.45
